# Special Issue: Gut Bacteria-Mucus Interaction

**DOI:** 10.3390/microorganisms7010006

**Published:** 2019-01-04

**Authors:** Nathalie Juge

**Affiliations:** Quadram Institute Bioscience, The Gut Microbes and Health Institute Strategic Programme, Norwich Research Park, Norwich NR4 7UA, UK; nathalie.juge@quadram.ac.uk

The mucus layer covering the gastrointestinal tract plays a critical role in maintaining a homeostatic relationship with our gut microbiota. The large intestine, which is home to most microbial species constituting the gut microbiota, is lined by a bi-layer of mucus. The outer layer provides a habitat for bacteria, whereas the inner layer maintains them at a safe distance from the epithelial surface. The terminal mucin *O*-glycans serve as attachment sites and metabolic substrates to the gut commensal bacteria, which have adapted to the mucosal environment. Technology progress has advanced our understanding of mucin biosynthesis, glycosylation and organization. However, the molecular mechanisms underpinning gut bacteria-mucin glycan interactions remain poorly defined. 

This issue gathers eight articles covering various mechanistic aspects of gut bacteria-mucus interactions and impact on health and disease. These include comprehensive overviews of the role of mucus in the interaction with the gut microbiota in humans [1] or in host-pathogen interactions during infections in farmed animals e.g. pigs, cow, chicken and fish [2]. Four papers specifically address bacterial adhesion to mucus and mucin glycans using different methodologies including atomic force microscopy (AFM) and optical tweezers (OT) [3], carbohydrate or mucin microarrays [4], and in vitro adhesion plate assays [5] or slot-blot analysis [6] using fluorescent-labelled microorganisms. These articles, focusing on probiotic species such as *Lactobacillus acidophilus* [5] or pathogens species such as *Helicobacter pylori* [4] highlight how the origin, chemical structure and glycosylation pattern of different mucins as determined by MALDI MS-MS analysis may affect recognition by pathogenic or commensal bacteria [6]. This adhesion is often driven by several mechanisms such as cell-surface glycans or proteins that act in parallel or in a consecutive manner [3]. Two articles provide further information on how diet including prebiotics [5], food additives or environmental pollutants [7] influence mucus-bacteria interaction and the role of cell-surface proteins in this interaction. One article is dedicated to a well-known mucin-degrader, *Akkermansia muciniphila,* revealing its occurrence in other anatomical regions of the gastrointestinal tract although its optimal ecological niche remains the mucus layer in the colon [8]. It is clear from this Special Issue that mucus-bacteria interactions are strain-, host- and niche-specific, and the result of an intricate interplay between the host local environment, pathogenic and commensal bacteria inhabiting this niche, and the diet. Collectively these articles stress the importance of bacteria-mucus interactions in influencing health or disease outcome in humans and animals. Gaining molecular knowledge in this field is warranted to provide better diagnostics and risk assessment and help design new probiotic or glycan-based nutritional strategies to improve human and animal health.
